# Framework for Guiding the Development of High-Quality Conversational Agents in Healthcare

**DOI:** 10.3390/healthcare11081061

**Published:** 2023-04-07

**Authors:** Kerstin Denecke

**Affiliations:** Institute for Medical Informatics, Bern University of Applied Sciences, Quellgasse 21, 2502 Biel, Switzerland; kerstin.denecke@bfh.ch

**Keywords:** conversational agent, chatbot, evaluation, quality, patient safety

## Abstract

Evaluating conversational agents (CAs) that are supposed to be applied in healthcare settings and ensuring their quality is essential to avoid patient harm and ensure efficacy of the CA-delivered intervention. However, a guideline for a standardized quality assessment of health CAs is still missing. The objective of this work is to describe a framework that provides guidance for development and evaluation of health CAs. In previous work, consensus on categories for evaluating health CAs has been found. In this work, we identify concrete metrics, heuristics, and checklists for these evaluation categories to form a framework. We focus on a specific type of health CA, namely rule-based systems that are based on written input and output, have a simple personality without any kind of embodiment. First, we identified relevant metrics, heuristics, and checklists to be linked to the evaluation categories through a literature search. Second, five experts judged the metrics regarding their relevance to be considered within evaluation and development of health CAs. The final framework considers nine aspects from a general perspective, five aspects from a response understanding perspective, one aspect from a response generation perspective, and three aspects from an aesthetics perspective. Existing tools and heuristics specifically designed for evaluating CAs were linked to these evaluation aspects (e.g., Bot usability scale, design heuristics for CAs); tools related to mHealth evaluation were adapted when necessary (e.g., aspects from the ISO technical specification for mHealth Apps). The resulting framework comprises aspects to be considered not only as part of a system evaluation, but already during the development. In particular, aspects related to accessibility or security have to be addressed in the design phase (e.g., which input and output options are provided to ensure accessibility?) and have to be verified after the implementation phase. As a next step, transfer of the framework to other types of health CAs has to be studied. The framework has to be validated by applying it during health CA design and development.

## 1. Introduction

When applying a conversational dialogue system or conversational agent (CA) in a healthcare setting, such agent is often used to mimic physicians or therapists, i.e., it is asking questions regarding the personal medical history, provides information on treatments or diseases, or suggests exercises for health and well-being. For the purpose of this paper, we consider health agents or CAs as digital health interventions that (1) have a software component accessible through a conversational user interface, (2) address a specific healthcare domain or process, such as medical history taking or delivering cognitive behavior therapy, and (3) aim at improving healthcare outcomes. We define an intervention as an activity undertaken to determine, prevent, improve, or stabilize a medical condition. Examples of health agents include agents for delivering cognitive behavior therapy [1], for collecting the medical history [2], or supporting medication management [3]. During the COVID-19 pandemic, many CAs have been released within a very short amount of time addressing a diverse set of use cases (e.g., risk assessment, information dissemination, surveillance) [4].

In contrast to general domain CAs, a CA applied in a healthcare context has to fulfill certain criteria: Supposed to be used by individuals suffering from a medical condition or in the need of help, health agents have to be safe in use to avoid patient harm, be tailored to the application area, use case and user’s context, and have to address data privacy and data security aspects [5]. To become accepted as treatments, health CAs are studied in clinical trials regarding efficacy, safety, or cost effectiveness [6].

It has been shown that rather technical aspects of health agents or aspects highly depending on their technical realization are not assessed and reported or only to a limited extent [7]. Denecke et al., for example, found out that aspects related to data privacy and security are rarely reported [8]. CAs often process personal identifiable information that are in protected healthcare settings. Thus, privacy and security are essential aspects to be able to successfully integrate health CAs in healthcare processes. A trusting relationship between the user and the agent is also necessary for health agents to pursue a user-specified goal (such as behavior change) and achieve a long-term commitment. In order to protect the user from being harmed by inappropriate, incorrect, or poorly presented information, the agent must also be accurate and understandable.

Proper functioning is also important when delivering a health intervention, thus, aspects related to the technical realization should be assessed before a health agent is tested at a larger scale in patients with health problems. In the context of evaluating mobile health apps, some tools like the Mobile App Rating Scale [9] or Health-ITUES have been suggested. Hensher et al. developed a mobile app evaluation framework comprising, among other things, interoperability, technical features, and developer credibility [10]. However, compared to mobile health apps, health CAs require additional criteria for evaluation given their focus on communication-based interaction, making these evaluation frameworks not really applicable. Since interaction with a health CA can only be realized by natural language, written or verbal, aspects related to language-based human–machine interaction are of relevance to be assessed before bringing systems into daily practice. Just imagine the frustration or even danger, when someone is in need of help and the CA is not understanding properly or the replies are not fitting the context.

Since the very beginning of the development of CAs, one direction of evaluating CAs was to study their human likeness using the Turing Test [11]. The idea was to measure if the CA is capable of making a human thinking that it is a human as well. Thus, this measure reflects the ability to imitate human behavior. Jadeja et al. distinguish four perspectives for evaluating general domain chatbots: Information retrieval (IR) perspective, User Experience perspective, Linguistic perspective, and Artificial Intelligence (AI) perspective [12]. Casas et al. [13] examined CA evaluation methodologies and assessed them according to the ISO 9214 concepts of usability. More specifically, they aligned the CA evaluation methods with the usability concepts of effectiveness, efficiency, and satisfaction. Concrete metrics and heuristics are not linked. For customer service CAs, several key performance indicators have been suggested by enterprises to measure the success of the CA [14,15]. Such indicators are only applicable to a limited extent to health CAs given the peculiarities described before and the particular usage scenario which differs significantly from customer service CAs. So far, a carefully designed, overarching framework for guiding development and evaluation of health CAs is still unavailable.

To address the unavailability of an evaluation framework for health CAs that fulfills the mentioned requirements, this work aims at providing guidance in terms of metrics, heuristics, and guidelines concerning quality of health CA. The primary audience of this paper are researchers and developers who design or develop health CAs. 

## 2. Materials and Methods

### 2.1. Preliminary Work towards the Framework 

**Figure 1:**
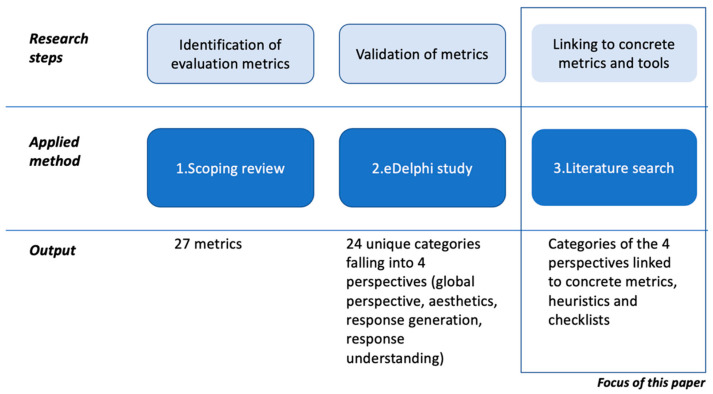
Three steps towards developing an evaluation framework for health CAs. With this paper, we build upon a scoping review that identified metrics and an eDelphi study that formed consensus on evaluation categories for health CAs.

The results of this work have been achieved through a longer process of research (see Figure 1). We conducted in previous work a scoping review to identify metrics that have been used to evaluate health CAs [16]. Afterwards, we formed a panel of experts working in the field of health CAs and found consensus regarding evaluation categories and metrics deemed relevant for health CA evaluation [6]. This work resulted in 24 evaluation categories grouped into four perspectives: global perspective, response understanding perspective, response generation perspective, aesthetics perspective.

Within the global perspective, we aggregated evaluation categories that are not related to a specific health CA component, but relevant for the system as a whole. Categories are related to the user interaction (ease of use, engagement, accessibility, flexibility in dialogue handling, task completion rate, error tolerance, dialogue efficiency) or to the overall quality of the health CA (context awareness, classifier performance, technical issues, security, content accuracy, speed). 

Response understanding refers to the CA’s ability to accurately interpret and understand the meaning of a user’s message. This involves analyzing the user’s input, determining the intent behind the message, and identifying any relevant information or context that may be needed to generate an appropriate response. Evaluation categories in the response understanding perspective are: understanding, concept error rate, word error rate.

The response generation component enables health CAs to engage in meaningful conversations with users and provide helpful and relevant information or assistance. The perspective response generation of the framework thus aggregates aspects or evaluation categories that ensure high quality responses, namely: appropriateness of responses, comprehensibility, clarity of speech, empathy, repetitiveness, realism, linguistic accuracy, speed of responses.

The aesthetics perspective comprises aspects regarding the visualization, i.e., font type and size, button color, shape, icon, background color, and content.

In previous work, we referred to the evaluation “categories” as metrics. Some of the categories were already concrete metrics (e.g., task completion rate), others were not (e.g., accessibility or security). In this work, we therefore decided to rename the previously called “metrics” to “categories”. Being aware that evaluation depends on the technical characteristics of the CAs under consideration, we identified archetypes of health CAs using a clustering approach [17]. For this work, we are focusing on the simplest types of health CAs, which are rule-based systems, also referred to as ad-hoc supporter [17]. They are based on written input and output, have a simple personality without any kind of embodiment. They run on a mobile device, are implemented as stand-alone software, and the interaction time is rather short.

In this work, we finalize the work towards the evaluation framework by suggesting concrete metrics and heuristics that specify a framework supporting health CA development and evaluation. The methodology comprises two phases: Phase 1 aims at identifying concrete metrics, heuristics, and checklists for the categories in the evaluation framework (details in Section 2.2), while in phase 2, a review of the results is conducted by experts in the field (details in Section 2.3). The underlying goal of this effort is to suggest a minimum set of evaluation metrics that is standardized. A standard set of metrics will allow for a comparison of health CAs in terms of quality; quality information could be reported along the categories of the framework. A framework could also provide a road map for conducting an evaluation and highlight aspects to be considered during development of a health CA. It specifies the data to be collected in terms of metrics and suggests tools to produce the data in an evaluation process. Several important requirements for an evaluation framework of health CAs exist [6]: It should (1) address issues specific for language-based interaction with a machine (response understanding, response generation), (2) address measures on a technical level (e.g., data privacy, security), and (3) address issues on a user level (ease of use, accessibility).

### 2.2. Phase 1: Identifying Concrete Metrics, Heuristics, and Checklists 

In phase 1, we linked the evaluation categories identified in previous work [6] to concrete metrics, heuristics, and checklists that are available in scientific literature, grey literature, or suggested by ISO recommendations in ISO/TS 82304-2:2021 Health software—Part 2: Health and wellbeing apps—Quality and reliability [18,19]. Based on our experience in health CA development and evaluation [2,3,8,16,17,20,21,22], items were selected and adapted if necessary. It is worth mentioning that the aim was not to list all possible metrics and checklists or heuristics, but to suggest a reasonable set that could become a minimal set of evaluation aspects and metrics to be considered for evaluation of health CAs.

### 2.3. Phase 2: Expert Review 

In phase 2, an expert evaluation was conducted to collect feedback on the metrics and heuristics gathered in phase 1. Participants were presented with the definition of the evaluation category, the set of metrics, heuristics, and checklists that were identified in phase 1, including the sources where these items were collected from. They were asked to rate and comment on the relevance of the linked metrics and heuristics for the evaluation of health CAs. Relevance was to be rated on a scale of −2 to 2 (being 2 the highest) to indicate how relevant each metric is for the evaluation regarding a concrete evaluation category. We excluded all items from the final framework where 60% or more than 60% of the experts voted with a value of {−2, −1, 0}. We recruited participants by contacting individuals that already participated in the Delphi study in which the dimensions of the evaluation framework were developed [6]. We provided them with an introduction letter and a link to the study. The questionnaire on general metrics was open for feedback for two weeks from 17–31 August 2022 while the questionnaire for the other metrics was open from 16–23 December 2022. Twelve persons were invited and five persons replied to our request, resulting in a return rate of 41.7%. Three persons (60%) had a work experience in the domain of health CA of 4–6 years; two persons (40%) had a work experience of 1–3 years in this particular domain. Regarding CA-related experiences, two persons developed a health CA; two supervised research on health CAs, and one person had a general interest in the topic of health CAs. Their educational background was medicine, psychology, and behavioral sciences (*n* = 1, 20%); health informatics, computer science, and engineering (*n* = 2, 40%); and computer science and engineering (*n* = 2, 40%). All five participants were male.

## 3. Results

**Figure 2:**
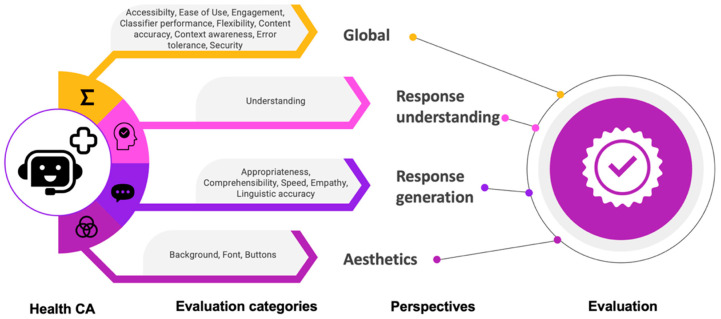
Evaluation framework for health CAs: It covers 4 perspectives with one or more evaluation categories.

In the following, we describe the metrics, heuristics, and checklists per category as a result of the two phases (see Figure 2 for an overview). Each section starts with the definition of the category, followed by a summary of the metrics or heuristics. As already mentioned, the original set of metrics mixed up categories and metrics of different granularities. There were already some concrete metrics included, which we now linked to some other categories. We consider speed, task completion rate, and dialogue efficiency as metrics of the category engagement and technical issues as metric of the category ease of use. Explanations will be given below. Additionally, clarity of speech (perspective response generation), word error rate, and concept error rate (perspective response understanding) are metrics for speech recognition and voice user interfaces and were excluded since we consider CAs with written input and output.

Table 1 shows the concrete metrics or checklist items for all nine categories of the global perspective. Table 2 summarizes the concrete metrics from the perspectives related to response generation, response understanding, and aesthetics. Additionally, we add tools for their assessment. Appendix A, Table A1 shows the relevance rankings per item of the experts, including mean values and variances among the judgements.

### 3.1. Evaluation Categories within the General Perspective

#### 3.1.1. Accessibility

Accessibility is the degree to which a person can use a health CA regardless of ability or disability [23]. As applied to health CAs, accessibility comprises two facets [24]:

- Accessibility of the service channel (e.g., font size, contrast, button size) can be addressed by providing multiple input or output modalities, ensuring adaptability of font size, sufficient contrast, as well as considering accessibility guidelines of the specific service channel.
- Accessibility of the conversation (e.g., use of language, confusing language, complex language, information overload) can be addressed by considering the readability and health literacy level of the health CA statements.

A number of scales and innovative machine learning approaches could be used for assessing the readability level of a health CA in order to match the readability to the level of expected users [25]. There are readability checkers available that implement, for example, the Flesch Kincaid Reading Ease Test or the Automated Readability Index (ARI). To assess the literacy level of the health CA (and their users), surveys and questionnaires assessing health literacy [24] (e.g., REALM-SF [26]) and e-health literacy [27] can be applied. 

For the framework, we came up with seven questions referring to these aspects, which should be considered in the design and implementation phase of the health CA (Table 1). Among other things they were derived from the Web Content Accessibility Guidelines WCGA 2.1 which were not developed particularly for health CAs, but for websites in general. Their consideration or compliance are recommended for health and wellness apps in the ISO/TS 82304-2 technical specification [18]. Translated to health CAs, we ask for hybrid interaction modalities to make CA content accessible to a broad range of users with possible disabilities. Beyond, it is crucial to consider the accessibility guidelines of the particular service channel through which the health CA is provided. A service channel could be a website, a mobile app, a messenger, etc. When implemented as part of a website or as component of an agent, User Agent Accessibility Guidelines (UAAG, https://www.w3.org/WAI/standards-guidelines/uaag/, accessed on 5 April 2023) have to be considered. These guidelines provide details of how to make user agents, such as browsers, media players, or other applications, that render web content, accessible to people with disabilities.

#### 3.1.2. Ease of Use

Ease of use refers to the extent to which a person believes that using a particular CA would be effortless. As metrics and heuristics, we suggest to quantify technical issues, to assess usability using a standardized tool (Bot usability scale), and consider heuristic criteria specifically developed for CA design. 

So far, methods for assessing ease of use of health CAs are manifold and are often build in an ad hoc manner instead of using well established tools [22]. The system usability scale [28] and Nielsen heuristics [29] are well-known means to study ease of use. Both have not been specifically designed for health CAs. We identified two methods that rely upon these two, but have been adapted to the peculiarities of CAs: Bot usability scale (BUS-11, [30]) and heuristics [31] for CA design. The set of heuristics for CA design comprises 11 criteria. They concern visibility of system status, match between system and the real world, user control and freedom, consistency and standards, error prevention, help and guidance, flexibility and efficiency of use, aesthetic, minimalist, and engaging design. They help users recognize, diagnose, and recover from errors, address context preservation, and trustworthiness (see Appendix A, Table A2, from Langevin et al. [31]). 

The BUS-11 comprises 11 items divided into five factors related to usability of a CA and to be assessed on a 5-point Likert scale (see Appendix A, Table A3): perceived accessibility to CA functions, perceived quality of CA functions, perceived quality of conversation and information provided, perceived privacy and security, time response. The items have been translated and validated already in four languages (English, Spanish, German, and Dutch). We believe it makes sense to rather use a usability scale adapted to the characteristics of a CA since such systems differ in the interaction from other information systems and an adapted version of a usability assessment tool considers this. 

Additionally, we suggest considering the metric technical issues—referring to the number of errors or glitches that occur while using a CA—under ease of use. When system errors or glitches occur, the ease of use is impacted. This is also consistent with the above mentioned adapted evaluation heuristics which include two heuristics related to errors (error prevention and help users recognize, diagnose, and recover from errors) [31]. It requires logging of errors, including type of error, e.g., critical service is down, database is down, required connection to the Internet is down. Further, appropriate error messages for the user have to be implemented, including user-friendly responses when errors occur or suggestions of alternatives in case of a critical situation (e.g., CA cannot understand, but user needs urgent support). 

#### 3.1.3. Engagement

Engagement concerns whether a user finds value in using a health CA and therefore continues using it [6]. Five metrics for engagement are suggested for our framework:

- Goal or task completion rate (the percentage of persons that complete a specific goal through the CA),
- Retention rate (proportion of users who have consulted the CA on repeated occasions over a given period),
- Speed (how quickly a session or task can be completed using a CA),
- Dialogue efficiency (length of the dialogue for solving a task),
- Satisfaction (whether the use of the health CA leads to a positive impact for a user).

We suggest considering speed and dialogue efficiency as metrics for engagement. Speed is defined as how quickly a session/task can be completed using a health CA and due to this definition, it relates to the task completion rate. Dialogue efficiency measures the length of the dialogue in the number of turns or the elapsed time [6]. Both metrics were already agreed to be relevant for health CA evaluation in a Delphi study [6].

Experts suggested to consider user satisfaction as part of the engagement. Therefore, we integrated the Net Promoter Score into our framework to judge satisfaction. It can be determined easily as part of usability studies. Dhinagaran et al. mentioned that the Net Promoter Score is a good indicator for satisfaction, i.e., users are asked whether they would be willing to recommend the CA to others [32]. It is comparable to determining a patient’s satisfaction with a healthcare professional: An individual recommends a particular healthcare professional when she is satisfied. 

#### 3.1.4. Classifier Performance

The category classifier performance gives insights on how well an algorithm performs in classifying data [6]. In a rule-based health CA—as it is considered here—we do not have any machine learning classifier integrated. Classifier performance could be interpreted as how well the rule-matching is performing given some user input. We can consider this an information retrieval task where, for some input query, the best fitting answer has to be identified. Standard metrics in this context are precision, recall, F-Score, and accuracy, which are included into the framework. 

#### 3.1.5. Flexibility in Dialogue Handling

Flexibility in dialogue handling concerns a health CA’s ability to maintain a conversation and deal with users’ generic questions or responses that are more, less, or different than expected [6]. Flexibility can be measured by counting conversation turns in the view of unexpected user input to find out whether the conversation could be concluded efficiently with a minimum of exchanges [33]. Further, some items of the TRINDI checklist [34] relate to the flexibility of dialogue. The TRINDI check list describes desiderata for evaluating dialogue systems. We suggest to consider relevant aspects from the TRINDI checklist to assess flexibility in dialogue handling. 

The TRINDI checklist consists of three sets of questions that are intended to elicit explanations describing the extent of a system’s competence. The first set consists of nine questions relating to the flexibility of a dialogue that the system can handle. The second set consists of five questions relating to the overall functionality of the dialogue system. The third set contains two questions relating to the ability of the dialogue system to make use of contextual/domain knowledge to provide appropriate responses to the user. The questions that make this list can be answered by: Y, yes; N, no; YN, partially; Y?, yes in theory; and ? not known. We suggest to consider the first eight questions of this checklist (see Table 1). 

#### 3.1.6. Content Accuracy

Content accuracy of health CAs is defined as proportion of responses that are consistent with clinical evidence. It includes correctness of triage and escalation strategies to be integrated in a health CA [6]. Measuring content accuracy is difficult, in particular when systems become complex and not all possible data entry options can be tested. Therefore, we recommend a content accurate-by-design process: To ensure accurate content, the underlying database has to be evidence-based and a maintenance process has to be in place, which is also a recommendation in the ISO/TS 82304-2 [18]. We encapsulated these aspects in several questions making up a checklist for content accuracy. In these questions, we are asking for the evidence-based knowledge base, for the maintenance process, for the involvement of relevant stakeholders in the development process (patient organization, healthcare professionals), and for information on the developer of the health CA. 

#### 3.1.7. Context Awareness

Context awareness refers to a CA’s ability to utilize contextual knowledge to appropriately respond to users. The context is naturally very important and key to a successful conversation. From the CA heuristics suggested by Langevin et al. [31], we derived two items that could be used to assess context awareness: Does the CA reliably recognize context switches? Is the CA able to clarify the context when it is not clearly formulated? Since health CAs might require health data from their users to make appropriate suggestions, an additional item added to the framework refers to the use of personal user data to contextualize answers or questions. 

#### 3.1.8. Error Tolerance

Error tolerance refers to a CA’s ability to detect and understand misspelled words in users’ replies. When a CA does not know the appropriate response, it posts fallback responses. Monitoring their rate of occurrence (i.e., the fallback rate calculated by dividing the number of times the CA had to fallback by the total number of messages the user asks in a conversation [35]) and the user messages that invoke them can help identify errors in natural language processing integrated in the health CA. 

#### 3.1.9. Security

The evaluation category security deals with how protected the CA is against hack attacks. The level of security required for a health CA varies on the functionalities and data that are processed. So far, no standards have been established and even reporting on security measures integrated into health CAs is limited [8]. To address security, the design process has to ensure already that the latest security protocols are used and security standards are fulfilled, i.e., a secure-by-design process is to be followed. We suggest to apply 11 aspects related to security from the ISO/TS 82304-2 guideline which were suggested to be considered when implementing health and wellness apps [19]. These 11 items can be used as a checklist when developing CAs in healthcare. We adapted them slightly to health CAs (see Table 1). Based on the expert feedback, two additional items were added to the checklist. One item refers to the availability of a data privacy statement; the other item concerns compliance with data privacy regulations of the different countries.

**Table 1:** Final set of metrics of the global perspective after expert assessment. “Design and implementation check” means that this has to be considered during the design and implementation of a health CA. The statements from “I”-perspective can be used as part of a user questionnaire to be answered on a 5-item Likert scale.

Category	Source of Metric or Heuristic	Metric	Tool or Phase Where the Metric Is Assessed
Accessibility	ISO/TS 82304-2 [18], WCGA 2.1, Lister et al. [24]	What is the readability level of the health CA content?	Readability checker, e.g., Flesch Kincaid Reading Ease Test or the Automated Readability Index (ARI).
		What is the health literacy of the user?	Health literacy surveys and questionnaires REALM-SF [26]) and e-health literacy [27]
		What is the required health literacy level for using the health CA?	Health literacy surveys and questionnaires REALM-SF [26]) and e-health literacy [27]
		Does the health CA provide alternatives for written in- and output (e.g., icons, images, voice as text alternatives)?	Design and implementation check
		Is the contrast between text and background color at least 4.5:1?	Design and implementation check
		Is it possible to resize the text?	Design and implementation check
		Are accessibility guidelines of the used service channel applied (e.g., WCAG, UAAG, Android/Apple Accessibility Guidelines)?	Design and implementation check
Ease of use	Review on Usability of CA by Denecke et al. [22], BUS-11 [36], Heuristics of CA evaluation: Langevin et al. [31]	Technical issues	Analysis of conversation protocols
		Usability assessment using the BUS as a standard means within CA evaluation	Exploratory user study with think aloud experiments or task-based evaluation
		Considering the suggested 11 heuristic criteria for health CA design	Design and implementation check
Engagement	Gan et al. [37] Key performance indicators [14,15]	Goal/task completion rate	Exploratory user study with think aloud experiments or task-based evaluation
		Retention rate	Analysis of conversation protocols
		Speed	Analysis of conversation protocols
		Satisfaction	Net Promoter Score
		Dialogue efficiency	Analysis of conversation protocols
Classifier perfor-mance		Precision	Analysis of conversation protocols
		Recall	
		F-Score	
		Accuracy	
Flexibility in dialogue handling	TRINDI Tick list [34]	Can the health CA deal with answers to questions that give more information than was requested?	Analysis of conversation protocols
		Can the health CA deal with answers to questions that give different information than was requested?	
		Can the health CA deal with answers to questions that give less information than was actually requested?	
		Can the health CA deal with negatively specified information?	
		Can the health CA deal with ‘help’ sub-dialogues initiated by the user?	
		Can the health CA reformulate an utterance on request?	
		Does the health CA deal with ‘non-help’ subdialogues initiated by the user?	
		Can the health CA deal with inconsistent information?	
Content accuracy	ISO/TS 82304-2 [18]	Is the underlying knowledge base evidence-based (e.g., appropriate peer reviewed scientific literature used)?	Design and implementation check
		Were healthcare professionals involved in the content development of the health CA?	
		Is there a maintenance process for the information included in the health CA?	
		Is information on the developer or content provider of the health CA provided?	
		Were patient organizations involved in the development of the health CA?	
Context awareness	Heuristics of CA evaluation: Langevin et al. [31]	Does the health CA reliably recognize context switches?	Analysis of conversation protocols
		Is the health CA able to clarify the context when it is not clearly formulated?	Analysis of conversation protocols
		Is the CA using personal user data to contextualize the request/question and generate the answers?	Design and implementation check, Analysis of conversation protocols
Error tolerance	Results from Delphi study [6]	Fallback rate	Analysis of conversation protocols
Security	ISO/TS 82304-2 [18], Langevin et al. [31], Denecke et al. [8]	Is ISO/IEC 27001 or another recognized standard related to information security management applied?	Design and implementation check
		Is an assessment of information security risks and potential consequences available?	
		Was a secure-by-design process pursued?	
		Are processes or measures in place for managing reliability and maintenance of third party software and components used in the health CA?	
		Is a process to prevent unauthorized access and modification to the source code and knowledge base of the health CA in place?	
		Is an information security policy available for the user?	
		Is the security of the health CA tested on a regular basis?	
		Is a process in place of reporting, identifying, assessing, logging, and responding to security vulnerabilities?	
		Is data encryption used for encrypting user data?	
		Is user authentication, authorization, and session management implemented?	
		Are standard operating procedures in place for processing personal identifiable information according to the privacy statement?	
		Is a privacy statement available for the user?	
		Is the health CA compliant with the current regulations about data privacy (e.g., GDPR in Europe and UK and HIPAA in US)?	

### 3.2. Evaluation Categories within the Response Generation Perspective 

Evaluation aspects in the response generation perspective include appropriateness of responses, comprehensibility, empathy, repetitiveness, realism, linguistic accuracy, and speed of responses. 

Appropriateness of responses is the proportion of appropriate responses of a CA to users’ questions or answers. This can be quantified by domain experts by manually analyzing the conversation protocols. Additionally, users can judge appropriateness of received responses after a CA conversation. Four statements from the BUS-11 [36] and some additional statements are considered relevant to assess appropriateness and are included in the framework. However, also according to the expert’s comments, it is difficult to define appropriateness of a CA’s responses since this can have multiple facets. Appropriate can mean “easy to understand”, “comfortable to talk with”, but also “solving my problem” or “solving my problem in an elegant way” (good language of use, easy to understand, friendly, empathetically). More research on this is still required. 

Comprehensibility, i.e., the degree to which a CA generates responses understandable by users, can be best judged by users. Three statements from the BUS-11 scale [36] were selected as metrics from the framework to assess comprehensibility. 

Realism is defined as how human-like a CA is. This is a subjective judgement which could be assessed using a single or multiple questions or assessed in interviews with users after interacting with the CA. We included the statement “My experience of talking to the CA was almost as real as talking to a real human.” to be judged on a Likert scale. However, the experts disagreed with this assessment of realism, and in their comments, all of them suggested to exclude the evaluation category realism. This led to the removal of realism as an evaluation aspect in this version of the framework. Typically, realism could be assessed using the Turing Test. In healthcare settings, however, it might be even important to let CA users know that they are talking to a machine instead of a human being. A user might be afraid to interact with a human-like bot or—from an ethical perspective—an inner bond of trust is generated which should not be built to not risk patient safety.

The category speed of response refers to the time a CA needs to respond to a user statement. This can be measured objectively using conversation protocols with time stamps and averaging the answer times from entire conversations. Additionally, a subjective judgement from the user on how they are perceiving the speed of responses is suggested. To cover this, we included one slightly adapted statement from the BUS-11 to subjectively assess speed of responses.

Empathy is a CA’s ability of understanding, being aware of, or being sensitive to feelings, thoughts, and experiences of a user based on their statements. Several empathy scores have been developed in the field of psychology (e.g., Empathy Quotient [38], Hogan’s Empathy Scale [39]). However, these questionnaires are comprehensive and may comprise 60 items to be answered. To facilitate the assessment, we suggest asking only three questions for subjective user assessment that reflect the user’s perceptions related to empathetical behavior of a health CA. Since the integration of sentiment and emotion analysis technologies in a health CA is a prerequisite for generating “empathetic” responses, one objective criterion asks for the availability of such technologies in the health CA under evaluation. 

Linguistic accuracy is defined as the proportion of linguistically correct responses. This comprises the grammatical structure of statements, correct use of terms such as pronouns or articles. We proposed the following objective metrics for linguistic accuracy:

- Percentage of grammatically incorrect sentences (e.g., wrong word order),
- Percentage of grammatically incorrect words (e.g., wrong flexion, wrong ending, spelling error),
- Percentage of wrong use of terms (e.g., wrong preposition, wrong pronoun).

We defined these percentages negatively, since it might be easier to count errors than counting correctly written words. These metrics can be determined using the conversation protocols from the human–CA conversations. A grammar and spell checker tool could be applied. For example, Python provides a library “LanguageTool”, which is an open-source tool used for grammar and spell-checking purposes, and it is also known as the spellchecker for OpenOffice.

In addition to these objective metrics, one statement from the BUS-11 is related to linguistic accuracy and was included for this evaluation category. This reflects the fact that the impact of linguistic accuracy to the user experience is subjective and depends on the educational level of users. 

### 3.3. Evaluation Categories within the Response Understanding Perspective

Within the perspective response understanding, understanding is of relevance to be assessed for rule-based systems with written input and output. Understanding refers to the system’s ability to adequately understand the verbal and nonverbal responses of users [16]. We suggest two questions that refer to response understanding to be answered by users after interacting with the health CA (see Table 2).

### 3.4. Evaluation Categories with the Aesthetics Perspective

Judging aesthetics is of qualitative nature and might be even conflicting with accessibility. For example, some buttons use effects that could also impact their readability and the background color could impact the contrast related to the text color. For the four aspects appearance of virtual agent, background color and content, font type and size, button color, shape, icon, we suggest conducting a user assessment with questions similar to the BUS-11 statements for sake of consistency. We found one study that evaluated background color and content, button color and shape [40]. However, the authors experimented with several versions and asked users for feedback. Since this procedure is very time consuming, we decided to include a simple question on this. 

**Table 2:** Final set of metrics for the perspectives related to aesthetics, response generation, and response understanding. The statements from “I”-perspective can be used as part of a user questionnaire to be answered on a 5-item Likert scale.

Category	Source of Metric, Heuristic	Metric	Tool
**Perspective Related to Response Generation**			
Appropriateness of responses	Results from Delphi study [6], Questions 6–8 from BUS-11 [36], expert feedback	Proportion of appropriate responses to users’ questions or answers	Analysis of conversation protocols
		I find that the CA understands what I want and helps me achieve my goal.	Exploratory user study with think aloud experiments or task-based evaluation
		The CA gives me the appropriate amount of information.	
		The CA only gives me the information I need.	
		I feel like the CA’s responses were accurate.	
		I feel like the CA’s responses (information) were adapted to my characteristics/conditions.	
		The CA gives me some relevant suggestions that provide me with additional relevant information.	
Comprehensibility	Results from Delphi study [6], Questions 3–5 from BUS-11 [36]	Communicating with the CA was clear.	Exploratory user study with think aloud experiments or task-based evaluation
		The CA was able to keep track of context.	
		The CA’s responses were easy to understand.	
Speed of response	Results from Delphi study [6], BUS-11 [36], expert feedback	Average time needed for a CA to post a reply.	Analysis of conversation protocols
		My waiting time for a response from the CA was aligned with my expectation.	Exploratory user study with think aloud experiments or task-based evaluation
Empathy	Results from Delphi study [6], Pinto et al. [41]	Does the CA include techniques for sentiment and emotion analysis?	Question to be answered by developer
		The CA appreciated what my experiences feel like to me.	Exploratory user study with think aloud experiments or task-based evaluation
		The CA did not realize how strongly I felt about some of the things we discussed.	
		The CA understood my words, but not the way I feel.	
Linguistic accuracy	Results from Delphi study [6], Question 9 from BUS-11 [36]	Percentage of grammatically incorrect sentences (e.g., wrong word order)	Analysis of conversation protocols (manually or using spell checker like LanguageCheck from Python)
		Percentage of grammatically incorrect words (e.g., wrong flexion, wrong ending, spelling error)	
		Percentage of wrong use of terms (e.g., wrong preposition, wrong pronoun)	
		I feel like the CA’s responses were accurate.	Exploratory user study with think aloud experiments or task-based evaluation
**Perspective related to response understanding**			
Understanding	Results from Delphi study [6], Pinto et al. [41]	I think the CA understood me.	Exploratory user study with think aloud experiments or task-based evaluation
		I think the CA usually understood all of what I said to him or her.	
**Perspective related to aesthetics**			
Background color and content	Results from Delphi study [6]	I like the background color.	Exploratory user study with think aloud experiments or task-based evaluation
Font type and size	Results from Delphi study [6]	The font type of the CA was well readable.	Exploratory user study with think aloud experiments or task-based evaluation
		The font size was appropriate to me.	
Button color, shape, icon	Results from Delphi study [6], Ali et al. [42]	I liked the button color.	Exploratory user study with think aloud experiments or task-based evaluation
		I liked the button shape.	
		Understanding the icons was easy.	

## 4. Discussion

### 4.1. Principal Findings

In this work, we suggested concrete metrics, heuristics, and checklists for evaluating health CAs from four perspectives: general perspective, response generation, response understanding, and aesthetics. Altogether, they form an evaluation framework to guide development and evaluation of health CAs. Metrics, heuristics, and checklists were identified from publications in the context of general domain CAs and mobile health applications as well as derived from expert’s experiences. The final framework considers nine aspects from a general perspective, five aspects from a response understanding perspective, one aspect from a response generation perspective, and three aspects from an aesthetics perspective (see Figure 2). All metrics included in the final framework were judged relevant for health CA evaluation by an expert group of five persons. They consist of subjective assessments from users and objective metrics that can be determined using the conversation protocols. Additionally, we provided suggestions for tools and methods to assess the metrics and heuristics, or suggested phases of the health CA development cycle, in which the aspects should be considered. 

### 4.2. Relation to Prior Work

Recently, the DISCOVER conceptual framework for design, development, and evaluation of health CAs [32] has been proposed. It distinguishes three stages: design, development, and evaluation of the health CA development, which are complemented by two cross-cutting considerations: user-centered design as well as privacy and security. In contrast to their work, we are suggesting a minimal set of concrete metrics for health CA evaluation. 

Similar to our work, Dhinagaran et al. claimed that evaluation of a health CA should start early in the development cycle [32]. It turned out, that some aspects of our evaluation framework, in particular the heuristics and checklists, should be considered already during the design and development phase (e.g., accessibility aspects, security). Regarding evaluation, Dhinagaran et al. distinguished usability testing (including assessment of usefulness and user experience), pilot and randomized trial for studying efficacy and effectiveness from assessing user engagement and acceptability [32]. As initially mentioned, we believe that technical aspects beyond usability have to be evaluated before conducting a trial. Regarding evaluating user engagement, they mention metrics, such as time spent interacting with the CA, number of times opened the app, usage duration. These metrics are similar to our suggested metrics retention rate or task completion rate. For the evaluation category engagement, our framework comprises four metrics. Adherence and acceptability—as suggested by Dhinagaran et al.—are not explicitly part of the set of metrics. We are suggesting instead the metric satisfaction which is connected to adherence and acceptability. 

Peras introduced a chatbot evaluation framework comprising five perspectives: user experience perspective, information retrieval perspective, linguistic perspective, technology perspective, and business perspective [43]. Some of the categories that are part of her framework are also included in our framework. However, the metrics suggested by Peras are often unspecific (e.g., general mentions of a rating scale, surveys, questionnaires as metrics for usability). Our framework goes beyond and suggests concrete metrics and questions to be answered.

Kaushik and Jones suggested a conceptual framework for the evaluation of conversational search interfaces [44]. Criteria include user experience, software usability, knowledge gain, and search experience. Conversational search interfaces differ from the type of CA we were considering in this paper. We recognized that some metrics are similar to the ones we identified (e.g., user engagement, response speed). However, health CAs require additional aspects to be evaluated in depth, such as security and content accuracy. 

Kowatsch et al. described an evaluation framework for digital health interventions, not specifically health CAs [45]. Their framework has similar evaluation categories as ours, e.g., ease of use, content quality, privacy, and security. Our category context awareness is related to their category of personalization. Their additional categories are more related to usage of the digital health intervention, e.g., adherence, perceived benefit, perceived enjoyment. Again, they are not suggesting concrete metrics, only the categories. It could be assessed whether some of the metrics we identified for health CAs could also be relevant for digital health interventions in general. However, we specifically selected metrics designed for CAs. 

For evaluating the quality of response generation in dialogue systems, multiple metrics have been suggested that require a reference corpus, including BLEU or ROUGE [46]. Their calculation requires reference corpora from human–human conversations and from human–CA conversations. Reference-free metrics compare the generated response in the context of the dialogue history [46]. Since the focus of this work is on rule-based dialogue systems where the pre-defined responses might only be slightly adapted during a conversation, we suggested a pragmatic way for evaluating appropriateness of responses. When adapting the framework to more complex health CA types, this aspect has to be re-considered. 

During the expert review, we received additional suggestions for extending the framework which we did not considered yet. One expert suggested the Acceptability E-Scale (AES, [47]) as a metric of engagement. This scale includes six items concerning ease and enjoyment of program use, understandability of questions, helpfulness for describing symptoms and quality of life, whether the amount of time to complete the program was acceptable, and overall satisfaction with the program. Since this scale has not been developed particularly for health CAs, we will access its applicability in future work. 

The first contact resolution rate was suggested as an additional metric for engagement by another expert. This metric originates from customer service and measures the percentage of users’ questions and requests solved at first contact. We believe application of this metric depends on the use case. A health CA that is supposed to collect information from the user might not be able to answer user questions, which prevents being able to use this metric. 

### 4.3. Implications for Health CA Development 

**Figure 3:**
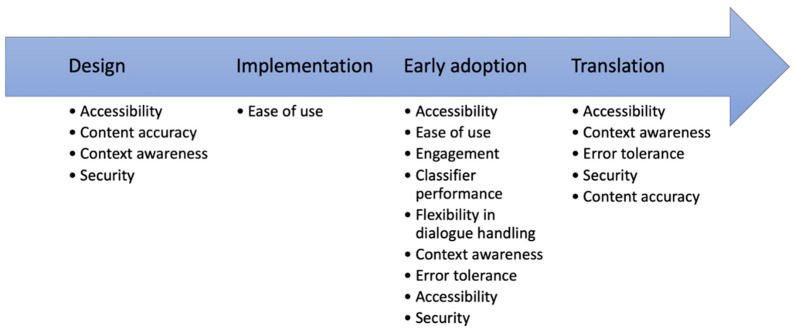
Evaluation categories along the health CA development path. Some aspects, e.g., accessibility, have to be considered or assessed in multiple phases.

The specification of the metrics, heuristics, and checklists for four evaluation perspectives demonstrates that evaluation aspects have to be considered repeatedly at the different phases of the health CA development (see Figure 3). This is reflected by the framework and was also the result from other related works [32,45]. In particular, aspects related to accessibility, content accuracy, or security have to be addressed in the design phase (e.g., which input and output options are provided to ensure accessibility?) and have to be verified after the implementation phase. We envision a phase where the health CA is used by early adopters. In this phase, the main aspects can be evaluated based on the interaction data (e.g., by analyzing the conversation protocols of the early adopters). A few aspects, such as security and content accuracy, should be assessed also after releasing the health CA to public. Since not all quality-relevant aspects can be completely quantitatively assessed with a reasonable effort (e.g., content accuracy), development of high-quality health CA requires also self-critical developers and health CA distributers (e.g., the suggested accurate by design approach). Guidelines for an “accurate-by-design” development of health CAs are still missing.

Our objective was to come up with a minimal set of metrics to achieve harmonization instead of suggesting all possible options for metrics and tools. This does mean, that health CA developers can decide to evaluate additional aspects related to the quality of their health CA or to study some of the aspects in more depth (e.g., the empathy). However, adopting our suggestions by all health CA developers would allow for a comparison and for ensuring a certain quality level. Our catalog of metrics should be also considered for reporting on the quality of a health CA. This would enable users and persons recommending health CAs to better judge a system, its reliability, and quality. 

Developers should also consider an analysis of conversation protocols for evaluation purposes. As Denecke et al. found out, this is rarely done [22] so far. As can be recognized from our framework, such analysis can be useful for assessing several of the evaluation categories and aspects.

### 4.4. Strengths and Limitations

This is, to our knowledge, the first evaluation framework specifically designed for health CAs and linked to concrete metrics, heuristics, and checklists. In this way, this work offers a clear comprehensive guideline of aspects to be evaluated during health CA development. The framework is based upon an analysis of evaluation metric literature as well as expert knowledge.

The linking was made based on the literature search and was confirmed by five experts. We recognized clear trends in their relevance voting. But it is clear, that five persons and the author of this paper cannot guarantee completeness. Further, we resisted on conducting another Delphi panel with a formal assessment of the judgements. We believe that it would be more valuable for the relevance of the framework when it is applied and tested during health CA design and development, i.e., the framework has to be challenged to study its feasibility and the applicability of the metrics and tools. We therefore decided not to do an expert assessment using a larger Delphi panel with more iterations. Instead, our vision is to ask the community to challenge the framework. For this purpose, we are currently preparing a website that will allow the community to comment on the evaluation metrics and categories. 

Out of the set of available tools, we sometimes had to decide to make a suggestion. For example, there are usability assessment tools that are similar to the selected BUS-11, such as the Chatbot usability scale [48]. We tried to select those that have already been adapted for health CAs or that seem to fit best in the context of health CAs. A limitation of this work is that we did not report on the reliability and validity of the suggested metrics and heuristics. We believe it is more valuable for the reader to go to the referenced papers than having a summary in this paper. 

## 5. Conclusions

Due to the lack of well-established evaluation guidelines for health CAs, the current work proposes a framework comprising concrete metrics, checklists, and heuristics for guiding development and evaluation of health CA. These metrics are considering four perspectives: a global perspective and perspectives related to response generation, understanding, and aesthetics [6]. The framework has been developed to form a minimal set of metrics that would allow to assess relevant quality aspects of health CAs. It is supposed to be useful to anyone who wants to develop a health CA for healthcare research or as health intervention. Specifically, we expect the framework to support in the following:

- Thoughtfulness on how to evaluate health CAs, which aspects to be considered within the development,
- Better navigating research and implementation choices by citations and pointers. It thus acts as a jumping off point for further reading,
- Moving towards comparison of quality of health CAs,
- Clearer reporting of quality aspects of health CAs.

In future work, the framework has to be validated by applying it as part of health CA development and evaluation. Feedback is requested by all researchers who apply the framework to update and improve it. We focused in this work on a specific type of health CA categorized as a rule-based ad-hoc supporter. Transfer of the metrics to more complex types of health CAs has to be assessed. Some metrics will be generalizable (e.g., ease of use or the heuristics). Others will have to be adapted or new ones will have to be integrated. This is especially true for evaluation categories and metrics from the language generation perspective. The main objective of the efforts towards an evaluation framework for evaluating high-quality health CAs is to guarantee patient safety for CA-based healthcare interventions as a result of carefully scrutinized health CAs. The variety of metrics suggests that an efficient approach for evaluating health CAs should be quite complex in order to provide quality assurance before clinical trials or wider implementation.

## Data Availability

The questionnaires are available at OSR (https://osf.io/mrxep/?view_only=8c76f1be1c63488f97be9f8c07764e25, accessed on 5 April 2023).

## Appendix A

**Table A1:** Results from the expert feedback. −2 = extremely irrelevant, −1 = irrelevant, 0 = neither relevant nor irrelevant, 1 = Relevant, 2 = Extremely relevant.

Category	Metric	−2	−1	0	1	2	Mean	Variance
Accessibility	What is the readability level of the health CA content?				20%	80%	1.6	0.3
	What is the expected health literacy level of the user?			40%	60%		0.6	0.3
	What is the health literacy level of the health CA?		20%	20%	60%		0.4	0.8
	Does the health CA provide alternatives for written in- and output?			20%		80%	1.6	0.8
	Is it possible to resize the text?			20%	20%	60%	1.4	0.8
	Is the contrast between text and background color at least 4.5:1?			20%	60%	20%	1	0.5
Ease of use	Technical issues				20%	80%	1.8	0.2
	Usability assessment using the BUS as a standard means within CA evaluation				80%	20%	1.2	0.2
	Considering the suggested 11 heuristic criteria for health CA design				100%		1	0
Engagement	Goal/task completion rate				80%	20%	1.2	0.2
	Activity volume			80%	20%		0.2	0.2
	Retention rate			20%	60%	20%	1	0.5
	Speed		20%		60%	20%	0.8	1.2
	Dialogue efficiency			40%	20%	40%	1	1
Classifier performance	Precision				40%	60%	1.6	0.3
	Recall		20%		20%	60%	1.2	1.7
	F-Score		20%		40%	40%	1	1.5
	Accuracy				40%	60%	1.6	0.3
Flexibilty in dialogue handling	Can the health CA deal with answers to questions that give more information than was requested?			40%		60%	1.2	1.2
	Can the health CA deal with answers to questions that give different information than was requested?		20%	20%	20%	40%	0.8	1.7
	Can the health CA deal with answers to questions that give less information than was actually requested?			20%	40%	40%	1.2	0.7
	Can the health CA deal with negatively specified information?				60%	40%	1.4	0.3
	Can the health CA deal with ‘help’ sub-dialogues initiated by the user?			20%	20%	60%	1.4	0.8
	Can the health CA reformulate an utterance on request?		20%		20%	60%	1.2	1.7
	Does the health CA deal with ‘non-help’ subdialogs initiated by the user?			40%	20%	40%	1	1
	Can the health CA deal with inconsistent information?				20%	80%	0.2	1.8
	Can the health CA deal with belief revision?			60%		40%	0.8	1.2
Content accuracy	Is the underlying knowledge base evidence-based?				40%	60%	1.6	0.3
	Were healthcare professionals involved in the content development of the health CA?				40%	60%	1.6	0.3
Context awareness	Does the health CA reliably recognize context switches?				40%	60%	1.6	0.3
	Is the health CA able to clarify the context when it is not clearly formulated?				60%	40%	1.4	0.3
Error tolerance	Fallback rate		20%		60%	20%	0.8	1.2
Security	Is ISO/IEC 27001 or another recognized standard related to information security management applied?	20%				80%	1.2	3.2
	Is an assessment of information security risks and potential consequences available?		20%		20%	60%	1.2	1.7
	Was a secure-by-design process pursued?		20%		40%	40%	1	1.5
	Are processed or measures in place for managing reliability and maintenance of third-party software and components used in the health CA?		20%		40%	40%	1	1.5
	Is a process to prevent unauthorized access and modification to the source code and knowledge base of the health CA in place?	20%				80%	1.2	3.2
	Is an information security policy available for the user?		20%			80%	1.4	1.8
	Is the security of the health CA tested on a regular basis?	20%		20%		60%	0.8	3.2
	Is a process in place of reporting, identifying, assessing, logging, and responding to security vulnerabilities?				40%	60%	1.6	0.3
	Is data encryption used for encrypting user data?	20%			40%	40%	0.8	2.7
	Is user authentication, authorization, and session management implemented?		20%		60%	20%	0.8	1.2
	Are standard operating procedures in place for processing personal identifiable information according to the privacy statement?			40%		60%	1.2	1.2
	Is a privacy statement available for the user?		20%		40%	40%	1	1.5
Appropriateness of responses	I find that the chatbot understands what I want and helps me achieve my goal					100%	2	0
	The chatbot gives me the appropriate amount of information.				100%		1	0
	The chatbot only gives me the information I need.			20%	80%		0.8	0.2
	I feel like the chatbot’s responses were accurate.			20%	60%	20%	1	0.5
Comprehensibility	Communicating with the chatbot was clear.			40%	40%	20%	0.8	0.7
	The chatbot was able to keep track of the context			20%	40%	40%	1.2	0.7
	The chatbot’s responses were easy to understand.				40%	60%	1.6	0.3
Realism	My experience of talking to the chatbot was almost as real as talking to a real human.	20%	20%	60%			0.2	1.2
Speed of responses	My waiting time for a response from the chatbot was short.			20%	40%	40%	1.2	0.7
Empathy	The chatbot appreciated what my experiences feel like to me.			20%	60%	20%	1	0.5
	The chatbot did not realize how strongly I fell about some of the things we discussed.		20%		60%	20%	0.8	1.2
	The chatbot understood my words, but not the way I feel.			40%	20%	40%	1	1
	Does the chatbot include techniques for sentiment and emotion analysis? (to be answered by developers)				60%	40%	1.4	0.3
Repetitiveness	Document similarity between chatbot protocols measured by cosine similarity over time.			80%	20%		0.2	0.2
Linguistic accurarcy	Percentage of grammatically incorrect sentences (e.g., wrong word order)				60%	40%	1.4	0.3
	Percentage of grammatically incorrect words (e.g., wrong flexing, wrong ending, spelling error)				60%	40%	1.4	0.3
	Percentage of wrong use of terms (e.g., wrong preposition, wrong pronoun)				40%	60%	1.6	0.3
	I feel like the chatbot’s responses were accurate.			20%	60%	20%	1	0.5
Understanding	I think the chatbot understood me.				40%	60%	1.6	0.3
	I think the chatbot usually understood all of what I said to him or her.				20%	80%	1.8	0.2
Background color and content	I like the background color.			20%	60%	20%	1	0.5
	I like the background content of the chatbot.		20%	60%		20%	0.2	1.2
Font size and type	The font type of the chatbot was well readable				40%	60%	1.6	0.3
	The font size was appropriate to me.				40%	60%	1.6	0.3
Buttons, icons, and shapes	I liked the button color.			20%	60%	20%	1	0.5
	I liked the button shape			20%	60%	20%	1	0.5
	Understanding the icons was easy.				40%	60%	1.6	0.3

**Table A2:** Heuristics of Langevin et al. [31].

Heuristics for CA	Explanation
Visibility of system status	The system should always keep users informed about what is going on, through appropriate feedback within reasonable time, without overwhelming the user.
Match between system and the real world	The system should understand and speak the users’ language—with words, phrases, and concepts familiar to the user and an appropriate voice—rather than system-oriented terms or confusing terminology. Make information appear in a natural and logical order. Include dialogue elements that create a smooth conversation through openings, mid-conversation guidance, and graceful exits.
User control and freedom	Users often choose system functions by mistake and will need an option to effortlessly leave the unwanted state without having to go through an extended dialogue. Support undo and redo.
Consistency and standards	Users should not have to wonder whether different words, options, or actions mean the same thing. Follow platform conventions for the design of visual and interaction elements. Users should also be able to receive consistent responses even if they communicate the same function in multiple ways (and modalities). Within the interaction, the system should have a consistent voice, style of language, and personality.
Error prevention	Even better than good error messages is a careful design of the conversation and interface to reduce the likelihood of a problem from occurring in the first place. Be prepared for pauses, conversation fillers, and interruptions, as well as dialogue failures, deadens, or sidetracks. Proactively prevent or eliminate potential error-prone conditions, and check and confirm with users before they commit an action.
Help and guidance	The system should guide the user throughout the dialogue by clarifying system capabilities. Help features should be easy to retrieve and search, focused on the user’s task, list concrete steps to be carried out, and not be too large. Make actions and options visible when appropriate.
Flexibility and efficiency of use	Support flexible interactions depending on the use context by providing users with the appropriate (or preferred) input and output modality and hardware. Additionally, provide accelerators, such as command abbreviations, that are unseen by novices but speed up the interactions for experts, to ensure that the system is efficient.
Aesthetic, minimalist, and engaging design	Dialogues should not contain information which is irrelevant or rarely needed. Provide interactional elements that are necessary to engage the user and fit within the goal of the system. Interfaces should support short interactions and expand on the conversation if the user chooses.
Help users recognize, diagnose, and recover from errors	Error messages should be expressed in plain language (no codes), precisely indicate the problem, and constructively suggest a solution.
Context preservation	Maintain context preservation regarding the conversation topic intra-session, and if possible inter-session. Allow the user to reference past messages for further interactions to support implicit user expectations of conversations.
Trustworthiness	The system should convey trustworthiness by ensuring privacy of user data, and by being transparent and truthful with the user. The system should not falsely claim to be human.

**Table A3:** BUS-11 items [36].

Factors	English Items
Perceived accessibility to chatbot functions	The chatbot function was easily detectable. It was easy to find the chatbot.
Perceived quality of chatbot functions	Communicating with the chatbot was clear. The chatbot was able to keep track of context. The chatbot’s responses were easy to understand.
Perceived quality of conversation and information provided	I find that the chatbot understands what I want and helps me achieve my goal. The chatbot gives me the appropriate amount of information. The chatbot only gives me the information I need. I feel like the chatbot’s responses were accurate.
Perceived privacy and security	I believe the chatbot informs me of any possibly privacy issues.
Time response	My waiting time for a response from the chatbot was short.
